# A systematic review of ergonomic and muscular strain in surgeons comparing robotic to laparoscopic approaches

**DOI:** 10.1007/s11701-025-02401-6

**Published:** 2025-05-31

**Authors:** Hedda Cooper, Hiu Ming Lau, Helen Mohan

**Affiliations:** 1 St Vincent’s Hospital Melbourne, Victoria, Australia; 2 Peter MacCallum Cancer Centre, Melbourne, VIC Australia

**Keywords:** Minimally invasive surgery, Traditional laparoscopic surgery, Robotic-assisted laparoscopic surgery, Muscular straining, Ergonomics

## Abstract

Robotic-assisted laparoscopic surgery has become more common in recent years with multiple benefits to patients. However, it poses musculoskeletal risks to surgeons, and given their long careers, it is vital to prolong surgeons’ longevity. This review aims to evaluate ergonomic impact and methods of ergonomic assessment in surgeons performing traditional and robotic-assisted laparoscopic surgery. To evaluate the ergonomics and how different body areas were being affected by robotic and laparoscopic surgeons, we performed a systematic review of studies following PRISMA guidelines and focussing on muscular and ergonomic assessment of laparoscopic surgeons. Electronic Ovid Medline and Embase databases were searched on the 15th of June 2023. 16 studies were identified, involving 508 surgeons. 530 traditional laparoscopies and 535 robotic-assisted laparoscopies were included. Mixed methods, including surface electromyography (sEMG) and Borg CR10 physical exertion scale, were used to assess muscular activation and fatigue. Whilst individual studies produced conflicting results, overall sEMG and BORG CR10 scales showed that in TLS the deltoid, triceps, biceps and wrist muscles are most commonly activated. In addition, in RALs, lower back, trapezius and finger muscles were activated most commonly. Muscle activations as a whole were generally lower in robotic-assisted laparoscopy. Survey tools such as NASA-TLX confirmed that overall RALS was less fatiguing than TLS for the majority of surgeons. This review explored the ergonomic risks faced by surgeons performing both traditional laparoscopic and robotic surgery. Further research, including standardised methodology and continuous ergonomic assessment, are warranted to ensure robotic surgery remains safe.

## Introduction

According to the International Ergonomic Association, ergonomics can be defined as the understanding of the interactions amongst humans and other elements of a system, and the optimisation of the environment to best fit a human work’s requirements [1]. A good ergonomic setting in the operating theatre should focus on how the working environment suits the working demands of surgeons, hence reducing unnecessary needs and burdens on surgeons’ bodies caused by prolonged standing and awkward posture [2]. Ergonomic measurement includes the alignment and angle of joints regarding the spinal upright position and posture. This is important because the mean operative time of surgeries is around 2 h, requiring surgeons to work in a fixed posture and limited area, which is prone to induce work-related musculoskeletal injuries [3, 4].

Surgical training programmes require significant financial and personal cost and effort; thus, prolonging the longevity of surgeons is important. The cost of becoming a surgeon involves different levels of expenses. Starting from medical school, it costs postgraduate domestic medical students around 10,000 AUD annually in a commonwealth-supported place for a 4-year medical course in 2014, which has been increased to 11,401 AUD annually in 2022 due to inflation and other cost factors from the course provider [5]. A study investigating the individual financial burden of surgical trainees in the UK and Ireland showed that the mean debt of medical graduates on acquiring a medical degree increased from £17,892.3 to £27,655.36 from 2000–2004 to 2010–2014, respectively [6]. Minimally invasive surgeries (MIS), including traditional laparoscopic surgery (TLS) and robotic-assisted laparoscopic surgery (RALS), provide numerous benefits to patients and surgeons, but it also puts surgeons’ health at risk. Ever since the introduction of TLS in the late 1990 s, it has been a preferred surgical modality to many patients by providing better postoperative outcomes, fewer surgical errors and intraoperatively complications, and better cosmetic results [7]. RALS, introduced in the 2000 s, further provides a more concise control for surgeons, and it was thought to improve surgeon’s posture and reduce musculoskeletal symptoms from lengthy operations [8]. However, a systematic review investigating the prevalence of the musculoskeletal disorder revealed that close to 74% of laparoscopic surgeons experienced the manifestations of the musculoskeletal disorder [9]. With almost three-quarters of laparoscopic surgeons experiencing musculoskeletal disorders, it has a huge impact on surgeons’ careers since it results in sick leaves, reduced caseload, early retirement and avoidance of specific surgical modalities [9]. Past literature studied the relationship between surgeons’ physical symptoms and various factors including the level of fitness, year of experience, age, height, hand dominance, gender and hand size, which showed that lower levels of fitness, being less experienced, younger in age and female surgeons correlated with the physical symptoms [10]. TLS and RALS instruments, including the laparoscope and the grasper handles, were designed to be one-size-fits-all, which also negatively impacted surgeons [9]. An understanding of how TLS and RALS directly affect different muscle groups in surgeons during surgery and how it correlates with the surgeons’ ergonomics is important. Past literature focussed on questionnaires to explore the affected body areas from surgeons’ subjective opinions but recently more studies explored intraoperative ergonomic assessment and objective measurement of muscle activity via the use of surface electromyography [11]. Specialties, such as general surgery, gynaecology, urology and colorectal surgery, utilising TLS and RALS heavily, have their unique surgical tasks and skills; thus, they face unique challenges in terms of muscular straining and ergonomics when performing surgery.

This study aims to evaluate the current literature to evaluate the ergonomic impact and methods of ergonomic assessment in the theatre environment of surgeons performing traditional and robotic-assisted laparoscopic surgery.

## Methods

**Fig. 1:**
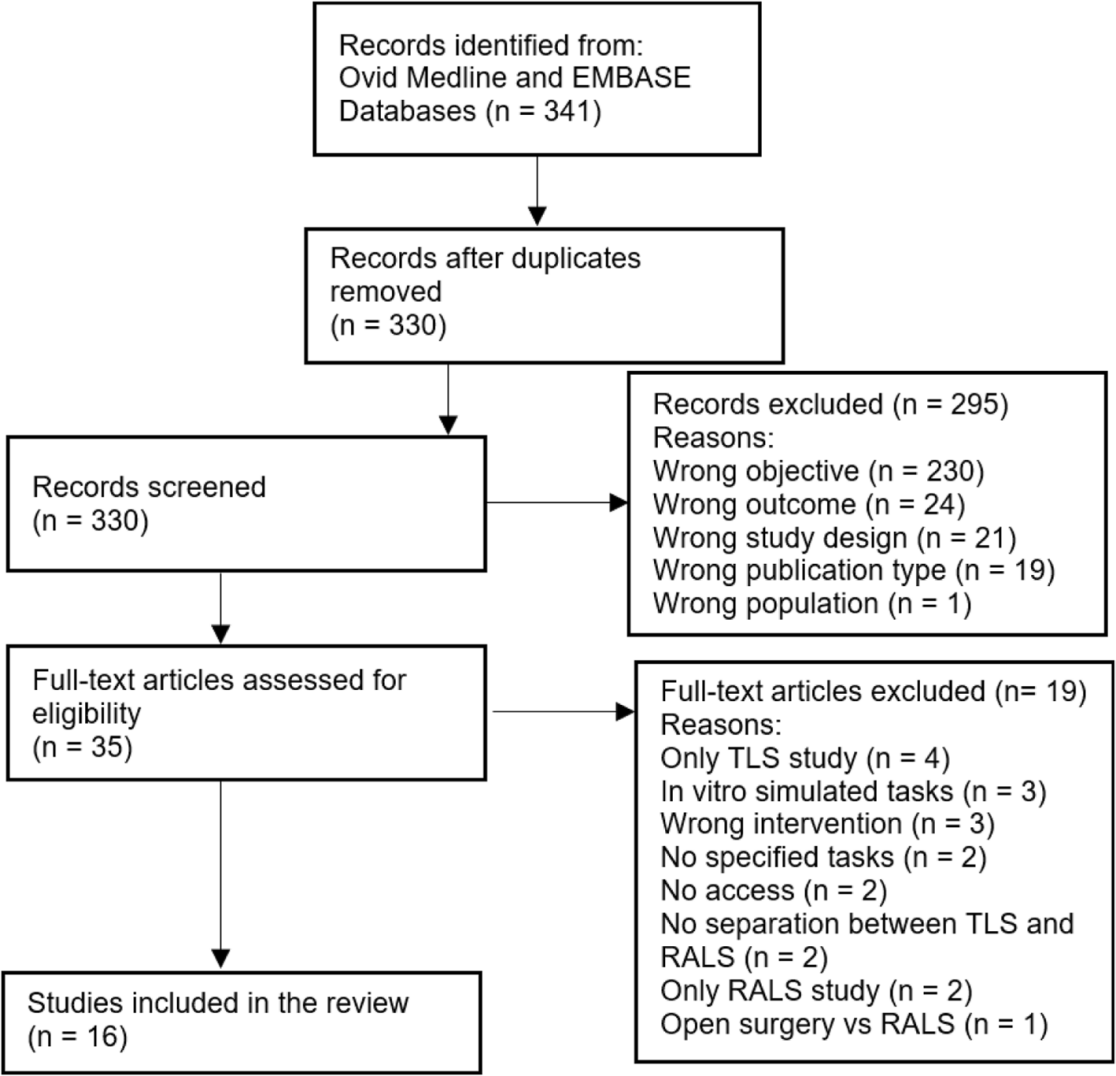
PRISMA flow diagram outlining the number of articles searched in both Ovid Medline and EMBASE on the 15 th of June 2023 and the final number of included articles with reasons for the excluded papers

A systemic review was conducted and reported in keeping with the PRISMA guidelines (Fig. 1). A meta-analysis was not suitable in this review due to the heterogeneity of outcome measurements. The search strategy was developed based on each element in the study aim, and Boolean operators were coupled with the search terms, as shown in Table 1. Electronic Ovid Medline and Embase databases were utilised to search for relevant literature on the 15^th^ of June 2023.

**Table 1:**
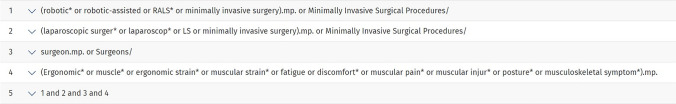
Search terms developed and utilised based on the study aim with Boolean operators on Ovid

Regarding inclusion criteria, only peer-reviewed articles that were published in the English language in the last 10 years were included. Papers focussed on the muscular straining and/or ergonomic assessment in laparoscopic surgeons performing TLS or RALS in patients were included. Objective or subjective and qualitative or quantitative measurements were included. All specialities were included.

Regarding exclusion criteria, protocols and review articles and papers with no relevancy were excluded. Papers with in vitro simulated tasks were excluded to ensure the studies reflect on the real-life theatre setting. Papers with no specified type of surgery were excluded. Papers that did not delineate TLS and RALS results were also excluded to ensure adequate evaluation and ability to distinguish specific muscle group activations between TLS and RALS. The study was not pre-registered with PROSPERO.

All studies found in the initial search were screened by two researchers based on the abovementioned criteria. The selected papers were analysed, and the basic demographics of the surgeons were compared. The methods used to assess the surgeon’s muscular straining and ergonomic measurement intraoperatively were assessed and compared. The outcome of the parameters tested was also extracted, analysed and compared.

## Results

The total number of the literature identified in both databases, Ovid Medline and EMBASE, was 341. 330 articles after the removal of duplication were screened based on their titles and abstracts. 35 articles were selected for full-text screening, and only 16 of the articles were included in the extraction and analysis. Reasons for excluding articles were detailed in the PRISMA flow diagram (Fig. 1). A summary of all studies’ demographics is listed in Table 2. 508 surgeons were studied in 15 studies, and no number of surgeons was available in J. Hotton et al. (2023) due to it only reporting the number of cases instead [12]. 535 RALS and 530 TLS were studied in 14 articles, whilst two articles were only questionnaire-based studies. 6 out of 16 articles mentioned the mean age of surgeons, and 9 out of 16 articles recorded the years of experience or the title of the surgeons. The number of articles studying gynaecology, urology, general surgery, colorectal surgery, paediatric surgery and bariatric surgery are 8, 4, 4, 5, 1 and 1, respectively.

**Table 2:** Demographics of 16 studies included in the review. The number of surgeons, the relevant number of cases, level of expertise, mean age, any preexisting health conditions, speciality, robotic surgical system and type of study are included. NM in the percentage of males is “not mentioned”

Publication [year]	Sample size [% of males]	Level of expertise	Mean of age	Preexisting musculoskeletal conditions	Specialty	Robotic system	Type of study
S. Anand et al. [2022][14]	1 surgeon [100%], 16 cases [11 RALS, 5 TLS]	Not mentioned	Not mentioned	Not mentioned	Urology	Not mentioned	Objective intraoperative observation
M. E. Tarr et al. [2015][28]	16 surgeons [25%], 86 cases [33 RALS, 53 TLS]	53% fellows, 34% residents and 13% attending surgeons	33	Not assessed	Gynaecology	Da Vinci Si and da Vinci S	Subjective survey pre- and postoperatively
G. P. Y. Szeto et al. [2013][20]	2 surgeons [NM], 4 cases [2 RALS, 2 TLS]	Not mentioned	Not mentioned	Not mentioned	Colorectal surgery	Da Vinci S	Objective intraoperative observation
A. M. Zihni et al. [2014][19]	1 surgeon [NM], 18 cases [5 RALS, 13 TLS]	Fellow	Not mentioned	Not mentioned	General surgery	Da Vinci Si	Objective intraoperative observation
B. Marcon et al. [2019][23]	5 surgeons [NM], 134 cases [65 TLS and 69 RALS]	Not mentioned	Not mentioned	Not mentioned	Urology	Da Vinci Si	Subjective intraoperative observation and subjective survey postoperatively
J. Hotton et al. [2023][12]	369 cases [193 TLS, 176 RALS]	Not mentioned	Not mentioned	Not mentioned	Gynaecology	Not mentioned	Subjective intraoperative observation and subjective survey postoperatively
P. R. Armijo et al. [2019][21]	16 surgeons [50%], 28 cases [18 TLS, 10 RALS]	Not mentioned	Not mentioned	No recent MSK condition	General surgery, Gynaecology, Urology and Colorectal surgery	Not mentioned	Objective intraoperative observation and subjective survey
V. Mendes et al. [2020][22]	24 surgeons [NM], 170 cases [88 RALS, 82 TLS]	13 Experienced [> 7 years of surgical experience],	Not mentioned	4 surgeons were reported to have MSK conditions	Urology [8], gynaecology [11] and paediatric surgery [3]	Da Vinci Xi	Subjective intraoperative observation and subjective survey postoperatively
		and 9 young surgeons					
K. Li-Jen et al. [2020][16]	1 surgeon [NM], 18 cases [10 RALS, 8 TLS]	Not mentioned	Not mentioned	Not mentioned	Colorectal surgery	Not mentioned	Objective intraoperative observation
S. Alsabah et al. [2019][24]	113 surgeons [94.7%]	19.4 years in medicine	45.2	52.3% had MSK injuries or conditions	Bariatric surgery	Not mentioned	Subjective survey
L. F. Grochola et al. [2019][26]	3 surgeons [NM], 60 cases [30 RALS, 30 TLS]	Not mentioned	Not mentioned	Not mentioned	General surgery	Da Vinci Si	Subjective survey postoperatively
T. Dalsgaard et al. [2020][13]	12 surgeons [41.7%], 24 cases [12 RALS, 12 TLS]	> 100 gynaecological cases	52	69% of surgeons reported having pain in the neck and lower back for the last 3 months	Gynaecology	Da Vinci Si	Objective intraoperative observation and subjective intraoperative observation
B. Kramer et al. [2022][17]	5 surgeons [40%], 40 cases [20 TLS, 20 RALS]	> 10 RALS cases, median 19 years in laparoscopic surgery	45	No recent MSK condition	Gynaecology	Da Vinci Si	Objective and subjective intraoperative observation and subjective survey intraoperatively
K. A. Butler et al. [2013][18]	6 surgeons [NM], 72 cases [16	4 attending surgeons, 2 fellows	Not mentioned	Not mentioned	Gynaecology	Da Vinci S	Objective observation and subjective survey
	TLS, 56 RALS]						
T. Dalager et al. [2020][15]	13 surgeons [92.3%], 26 cases [13 TLS, 13 RALS]	> 6 years of surgical experience	49	No recent MSK condition	Colorectal surgery	Not mentioned	Objective intraoperative observation and subjective survey
A. Alhusuny et al. [2021][25]	290 surgeons [52.4%], 59 constantly doing RALS	81% consultants or fellows	46.2	18% had MSK issues in the last 7 days before commencing the survey	76.6% gynaecology, 13.8% general surgery, 5.5% colorectal surgery	Not mentioned	Subjective survey

### Methods of muscular assessment

The commonest methods studies used to assess muscle group activity were surface electromyography (sEMG) and Borg CR10 physical exertion scale. sEMG was used to assess the percentage of the maximal voluntary contraction force of a particular group over the surgical time, whilst Borg CR10 physical exertion scale was used to assess 7 body parts: fingers, wrist, lower arm, neck, shoulders, low back and legs by giving a score from 1 to 10 from the surgeons during the surgery every 30 min or 60 min [13]. Rapid Entire Body Assessment (REBA) and Rapid Upper Limb Assessment (RULA) were also used in two studies for ergonomic assessment [14, 15]. Other tests including the Purdue Pegboard test, handgrip test, body posture observation and gravimetric position sensor were also used in individual studies [13, 15–18].

### sEMG

As shown in Table 3, 6 studies utilised sEMG on surgeons, and the results were conflicting. Major muscle groups that are used in laparoscopic surgery were identified and assessed, including the cervical erector spinae for extension and flexion of the spine, trapezius muscle for head and shoulder movement, deltoid for the abduction of the shoulder, biceps and triceps for the flexion and extension of the elbow joint, and forearm muscles for the wrist/finger flexion and extension. Zihni et al. [2014][19] and Dalsgaard et al. (2020) [13] both found that surgeons in the RALS modality had a lower muscle activation in all assessed muscle groups, including forearm, elbow, shoulder and neck, except Dalsgaard et al. (2020) [13] found that surgeons in RALS utilised the lower back muscle more than TLS. Dalager et al. (2020) [15] found that surgeons in RALS activated their muscles less than surgeons in TLS, except for the left trapezius muscle, whereas Kramer et al. (2022) [17] found that surgeons in RALS utilised less in the trapezius muscle but more in the extensor muscle of the wrist. Szeto et al. (2013) [20] indicated a conflicting result in the study where the trapezius muscle was activated more in one surgeon in RALS and the same result in the other surgeon in TLS. Armijo et al. (2019) [21], a study mixing different specialties, also found that all muscle groups assessed were activated less in the TLS group.

**Table 3:** A summary of 13 studies that utilise intraoperative measurement to assess muscular straining and ergonomics from performing the surgery. Only statistically significant results were summarised, and surgical tasks were detailed if they were provided in the paper

Publication [year]	Study design	Surgical tasks	Muscular straining measurement	Ergonomics measurement	Result
S. Anand et al. [2022][14]	Objective intraoperative observation	Ureteric reimplantation	N/A	Rapid Entire Body Assessment [REBA] tool, a validated tool to assess for neck, trunk, leg, upper arm, lower arm, wrist postures, load score and coupling. Two individual assessors were present to assess the surgeon’s posture	Two REBA assessments were done during the surgery at the point of the most complex steps, ureteral dissection and tunnel creation The surgeon experienced a medium ergonomic risk in RALS compared to a very high ergonomic risk in TLS. The scoring in RALS mostly came from trunk flexion [0° to 20°], neck flexion [0° and 10°], lower arm flexion [60° and 100°] and wrist flexion and extension [0° and 15°]. The scoring in TLS mostly came from the twisting neck with flexion [0° and 20°], trunk flexion [0° to 20°] with side bending, raised upper arm [20° to 45°], lower arm flexion [0° to 60°] and twisting wrists with an angle of > 15°
G. P. Y. Szeto et al. [2013][20]	Objective intraoperative observation	Low anterior resection	Surface electromyography [sEMG] on cervical erector spinae, upper trapezii and anterior deltoid on both surgeons. Wrist flexors and extensors were only recorded on one surgeon Only 1 h of the rectal mobilisation was recorded	N/A	A conflicting result was observed that one surgeon was experiencing a higher muscle activity in the left upper trapezius in the robotic surgery and the other surgeon was having a higher muscle activity in both upper trapezii in the laparoscopic procedure. With the robotic surgery alone, there was high muscle activity recorded in both flexor carpi ulnaris and extensor carpi ulnaris
A. M. Zihni et al. [2014][19]	Objective intraoperative observation	Complex foregut procedures for RALS and abdominal wall hernia for TLS	sEMG on bilateral biceps, triceps, deltoid and trapezii muscles	N/A	Bilateral biceps, triceps and deltoids were found to be significantly activated in surgeons performing traditional laparoscopic surgery. No difference in activity was found in the trapezius muscle between traditional laparoscopic and robotic surgery. No data were collected on the forearm and thenar muscles
B. Marcon et al. [2019][23]	Subjective intraoperative observation	Nephrectomy	Borg CR10 physical exertion scale, every 30 min, on seven body parts, neck, right shoulder and arm, left shoulder and arm, right forearm and wrist, left forearm and wrist, legs and lower back	N/A	There were significant results that the left shoulder and arm were less exerted in robotic-assisted surgery when the task was prolonged for more than 150 min. The left forearm and hand were also less exhausted in robotic-assisted tasks from 30 to 150 min. The right shoulder and arm were also less exerted at two time points, which were 150 and 210 min, in robotic-assisted surgery However, the lower back was more stressed in robotic-assisted surgery at all time points
J. Hotton et al. [2023][12]	Subjective intraoperative observation	Total hysterectomy/radical hysterectomy/pelvic lymphadenectomy/paraaortic lymphadenectomy	Borg CR10 physical exertion scale, every 60 min	N/A	Besides the back region, all other body parts were more exhausting in TLS than in RALS overall. In TLS, surgeons developed more fatigue in the hands, arms, necks and legs over surgical time, however, surgeons performing RALS did not report such findings
P. R. Armijo et al. [2019][21]	Objective intraoperative observation	8 foregut, 8 hernia repairs, 4 bariatric surgeries, 3 obstetric/genealogical surgeries, 1 kidney transplant, 1 hepatectomy and 1 rectopexy	sEMG on upper trapezius, anterior deltoid, flexor carpi radialis and extensor digitorum	N/A	The upper trapezius, anterior deltoid and flexor carpi radialis were more activated in robotic surgeries. In addition, the median frequency level for the extensor digitorum was recorded to be higher in the TLS, indicating a higher fatigue level. Overall, the median frequency level of the upper trapezius and anterior deltoid was lower than the flexor carpi radialis and extensor digitorum in both modalities
V. Mendes et al. [2020][22]	Subjective intraoperative observation	Surgeries in urology, gynaecology and paediatrics	Borg CR10 physical exertion scale, every 30 min	N/A	Physical discomfort experienced by the surgeons was significantly higher over time in the TLS than in the RALS in all 7 body parts. The forearm and back were affected the most in the TLS. In the meanwhile, the discomfort was not changed in all 7 body parts over time in RALS, except for the back. There was no difference when factoring in the experiences of surgeons
K. Li-Jen et al. [2020][16]	Objective intraoperative observation	5 hemicolectomies, 2 subtotal colectomies and 1 low anterior resection in TLS. 5 low anterior resections, 1 anterior resection, 1 radical proctectomy and 3 transanal surgeries in RALS	Purdue Pegboard test to assess hand dexterity and handgrip test for strength. Retested when 2 h into the surgery	N/A	There were no significant differences in both hand dexterity between RALS and TLS pre- and post-surgery. There was a significant decrease in hand strength in TLS after 2 h of the procedure, indicating significant muscle fatigue Within the RALS group, there was no difference in both dexterity and muscle fatigue pre- and post-surgery, suggestive of a better ergonomic environment
L. F. Grochola et al. [2019][26]	Subjective survey postoperatively	Single-port laparoscopic cholecystectomy and robotic cholecystectomy	A modified local experienced discomfort score and subjective mental effort questionnaire after performing the procedures	N/A	Robotic cholecystectomy showed generally better advantages for surgeons in both mental and physical aspects. There was a statistically significant result that robotic surgery lowered the mental burden on surgeons, and the same trend was observed for physical discomfort, however, it was not significant
T. Dalsgaard et al. [2020][13]	Objective intraoperative observation and subjective intraoperative observation	Hysterectomy	sEMG on extensor carpi radialis, flexor carpi radialis, neck extensor muscles, upper trapezius muscles and erector spinae muscle Borg CR10 physical exertion scale on fingers, wrist, lower arm, neck, shoulders, lower back and legs for perceived rating	Body posture observation for neck, shoulder, hand, wrist, back, legs and feet	Based on the sEMG results, neck and shoulder muscles were utilised less heavily in RALS than in TLS, with a 33% to 61% difference in the level of static measurement. The highest neck muscle activation was also observed in the TLS compared to RALS. On the dominant side, the wrist extensor muscle was activated more heavily in TLS than in RALS
					However, low back muscles of the dominant side were utilised more, on all static, average and mean measurements, in RALS, compared to TLS With TLS, shoulder muscles were also activated more on the dominant side of the surgeons, in couple with the uneven weight distribution due to the nondominant foot being on the pedal from the body posture observation On the Borg CR10 scale, surgeons reported generally less exertion in the RALS, significantly in the legs, dominant shoulder and finger regions Regarding the posture observations, it was not mentioned how it was done or who assessed the surgeons. However, a few issues were noticed in it. Within TLS, surgeons usually abducted their dominant arm for a long time without switching positions or taking a break They also twisted their trunk to compensate for the need to put the nondominant foot on the pedal, causing uneven distribution of weight bearing Within RALS, as procedures went on, surgeons tended to forget to use the armrest and the chair was further away from the robotic console. Both could lead to a worsening muscular straining of the shoulder and arm
B. Kramer et al. [2022][17]	Objective and subjective intraoperative observation and Subjective survey intraoperatively	32 Hysterectomies, 8 subtotal hysterectomies with cervical-colposacropexy	sEMG on trapezius muscles, lower arm extensor digitorum muscles and flexors carpi radialis muscles. An 11-point Likert scale was used within the procedure every 20 min to test for physical discomfort	Gravimetric position sensors, recording inclination angles against the normal	Regarding sEMG, trapezius muscles were not utilised as much, reduced in the time of static activation and increased in resting time in RALS than in TLS. A similar observation was made for the flexors carpi radialis muscles. However, extensor digitorum muscles were more activated in the static component in the RALS Regarding ergonomic postures, neck and torso flexion was observed to be to a higher degree in surgeons in RALS than those in TLS. There was a decrease in arm abduction and anteversion in RALS as well
K. A. Butler et al. [2013][18]	Objective observation pre- and postoperatively and subjective survey pre- and postoperatively	The main procedures were hysterectomy and oophorectomy	Hand grip dynamometer, the surgeon’s dominant hand maximum strength was assessed for 30 s pre- and post-procedures Single-leg standing test was also assessed for postural stability VAS was also used to analyse the fatigue of a surgeon after a procedure	N/A	There was no difference in the duration of the surgeries Surgeons had worse postural stability after performing TLS than after performing a RALS. There was no significant finding in the difference in fatigue between groups from the grip strength test. In addition, there was no significant finding in the VAS on fatigue as well between groups
T. Dalager et al. [2020][15]	Objective intraoperative observation and subjective survey pre- and postoperatively	Different types of bowel resections and anastomosis formation	sEMG on extensor carpi ulnaris, extensor carpi radialis longus, flexor digitorum superficialis, upper trapezius and upper neck extensor	Postural observation every 10 min by Rapid Upper Limb Assessment [RULA] tool	Before the start of the study, over 69% of surgeons reported having preexisting muscular pain predominantly in the neck and lower back for the last 3 months from operating Regarding the sEMG, the right forearm flexor muscle has a higher muscular activation on the static level in TLS than in RALS. All forearm muscles also were recorded to have higher muscular activity in TLS than in RALS. Right forearm muscles were also observed to be performing longer but weaker activities in RALS whilst they performed stronger but shorter activities in TLS. However, the left upper trapezius was recorded to have a higher reading in the peak measurement in RALS than in TLS
			Perceived physical exertion assessed by Borg CR10 scale before and after surgery		There was no difference found for the neck muscle Within TLS, there were also uneven muscle activation readings amongst body sides, with higher activation readings on the right side Regarding the RULA assessment, the surgeon’s posture was graded from 1 to 4 after the assessment. In the TLS group, over 70% of the observations graded 3 or 4, indicating the need for change or implement changes, respectively. In the RALS, with at least 80% of observations graded to be 2, indicating changes might be needed. There were also significant findings that TLS surgeons performed worse in the right upper extremities, neck, trunk and leg regions than RALS surgeons. However, the RALS surgeon’s postures were graded worse in the left upper extremities Regarding perceived physical exertion, there was no significant difference between modalities before and after surgery

### Borg CR10 physical exertion scale

As shown in Table 3, 5 studies used the Borg CR10 physical exertion scale for surgeons to subjectively rate their body discomfort during the surgical modalities. Most studies shared the same findings that RALS surgeons felt less fatigue in all body parts than TLS surgeons, except for the lower back where RALS surgeons felt worse with long surgical time. Only Dalager et al. (2020) found that there was no statistically significant difference between the modalities [15].

### Other tests for muscular straining measurement

Li-Jen et al. (2020) reported that there was no difference in hand dexterity between RALS and TLS with the Purdue Pegboard test; however, the grip strength test showed that TLS surgeons displayed significant fatigue compared to RALS surgeons [16]. However, K. A. Butler et al. (2013) found no difference in the hand grip strength test [18].

### Other ergonomics measurements

Anand et al. [2022] used REBA and found that the RALS modality had an ergonomics advantage over TLS; however, both were at risk for further improvement in terms of ergonomic setting [14]. On the other hand, Dalager et al. [2020] used RULA and found the same type of result as Anand et al. [2022], with an emphasis on the worse ergonomic positions in neck, and leg regions in TLS surgeons [14, 15]. With the body posture observation utilised by Dalsgaard et al. [2020], prolonged abducted dominant arm and a twisted trunk for the pedal were the striking features in TLS surgeons, whereas the underutilisation of the armrest and chair not being close to the console were the striking features in RALS surgeons [13]. Kramer et al. [2022] with the use of gravimetric position sensors found similar results that there was an increase in arm abduction in TLS surgeons and an increase in trunk and neck flexion in RALS surgeons [17]. As shown in Table 4, with studies that used questionnaires, 5 out of 8 utilised the National Aeronautics and Space Administration Task Load Index [NASA-TLX] postoperatively. Piper Fatigue Scale-12, body part discomfort and customised surveys were also used to assess different aspects of surgeons experiencing physical symptoms.

**Table 4:** A summary and result of 8 studies that utilise quantitative and qualitative surveys to assess surgeons’ subjective feelings after surgeries and how TLS and RALS affected their physical symptoms

Publication [year]	Types of survey	Survey result
M. E. Tarr et al. [2015][28]	Quantitative and qualitative, the Body Part Discomfort [BPD] survey and the NASA-TLX	Survey scores were compared pre- and postoperatively. BPD survey and NASA-TXL results revealed no statistical significance. Combining both the BPD survey and NASA-TXL results, surgeons experienced less discomfort in the lower neck, shoulder and back region in the robotic setting than in the laparoscopic setting after surgery
B. Marcon et al. [2019][23]	Quantitative and qualitative, NASA-TLX	Physical demand was scored lower in robotic-assisted surgery than in laparoscopic surgery whereas personal performance was also higher in the RALS group. However, temporal demand and the feeling of frustration were scored higher in robotic-assisted surgery than in laparoscopic surgery
J. Hotton et al. [2023][12]	Quantitative and qualitative, NASA-TLX	Surgeons reported that RALS was less physically demanding than TLS, both in ability and activity, however, they felt that they performed better in the TLS. There was no difference in other domains including mental demand, frustration and required effect input
P. R. Armijo et al. [2019][21]	Quantitative and qualitative, the Piper Fatigue Scale-12	4 areas were analysed in the survey, including behaviour, affection, cognition and sensory. The only difference was that the fatigue level increased after procedures in both laparoscopic and robotic arms. Overall, no other difference was detected
V. Mendes et al. [2020][22]	Quantitative and qualitative, NASA-TLX	Surgeons were asked to fill in the survey right after the procedures. In both experienced and young surgeons, there was a higher global workload in RALS, compared to TLS. At the same time, performance was better in the TLS arm, compared to the RALS arm, from the perspective of surgeons. Amongst young surgeons, effort, mental and physical demands were higher in RALS than in the TLS. Amongst experienced surgeons, only physical demand and performance were higher and worse respectively in RALS, than in TLS. With the surgeons that performed both modalities, RALS provided a better environment in 6 areas, physical and mental requirements, effort, performance, frustration and overall workload
S. Alsabah et al. [2019][24]	Quantitative and qualitative surveys focus on the physical discomfort of different body parts and how it affects bariatric surgeons	It is worth noticing that nearly 95% of respondents were male. Two-thirds of surgeons experienced some form of physical discomfort related to performing surgery, and over a quarter of those reduced caseload due to that. Regarding the body areas being affected the most, surgeons experienced more discomfort in the back, shoulder and back, and neck in open surgery, TLS and RALS, respectively. Close to 30% of the surgeons felt that the discomfort had affected their performance. It was also shown that female TLS surgeons had a higher prevalence of pain in different areas. The supine patient position was also associated with more discomfort in the wrists, whilst the French position was not moderate physical activity showed a protective role in the amount of physical discomfort a surgeon experienced. With close to no and over 3 h of exercise each week associated with an increased amount of physical discomfort over 57% of surgeons sought medical help with their physical discomfort and physical discomfort was self-resolved in around 55% of those. Surgical intervention was needed in 6% of surgeons experiencing physical issues
B. Kramer et al. [2022][17]	Quantitative and qualitative, NASA-TLX	Surgeons in RALS experienced fewer physical demands but higher mental demands than those in TLS
A. Alhusuny et al. [2021][25]	Quantitative and qualitative survey with 52 items, focussing on the prevalence of physical symptoms and visual symptoms, and association with individual and workplace factors	This study targeted the Australian surgeon population. The prevalence of work-related physical symptoms was found to be two-thirds of the surgeons, mainly affecting their neck or shoulder and/or visual symptoms. Around one-third of surgeons reported having some variety of neck or shoulder pain whilst another one-third of surgery reported having visual symptoms during procedures, with dry eyes, blurry vision and difficulty focussing as the main complaints. Around 18% of the surgeons reported having both issues Around the workplace factors, over 50% of the TLS surgeons would adjust the height of the table and position of the foot pedals and monitors for a better ergonomic environment. In addition, 60% of the TLS surgeons also adjusted the light level in the procedure. 59% of the surgeons also reported having asymmetrical weight bearing which could lead to the manifestation of physical symptoms, followed by forward head movement [36%] and shoulder elevation [33%]. Amongst the robotic surgeons, only 24% of them would adjust console seat height and around 15% would change the feet or torso positions For TLS, there was an association in prevalence and severity between visual symptoms and neck or shoulder issues. Workplace factors, including forward head movement, uneven weight bearing, adjusting the temperature of the theatre, and being female, were also associated with worsening neck or shoulder problems and visual issues. Wearing a pair of spectacles also correlated with visual symptoms Regarding RALS, none of the aforementioned factors was associated with the worsening of either physical symptoms or visual symptoms

### NASA-TLX results

6 main areas, mental demand, physical demand, temporal demand, performance, effort and frustration were assessed in the survey. Out of the five studies, there were few similar results. Three studies found that surgeons felt RALS was less physically demanding. However, Hotton et al. [2023] and Mendes et al. [2020] found that surgeons felt like they performed better in the TLS [12, 22]. Marcon et al. [2019] found that RALS surgeons felt more frustrated and had a higher temporal demand [23]. Kramer et al. [2022] revealed that RALS is more mentally demanding than TLS for surgeons [17]. Mendes et al. [2020] also found that it required more effort and was mentally demanding for young surgeons in the RALS arm; however, when comparing between modalities in surgeons that performed both, it was found that RALS was superior in all 6 areas [22].

### Other surveys

With the Piper Fatigue Scale-12 that investigated 4 domains, there was no difference between modalities and the only significant difference before and after surgeries was a higher fatigue level [21].

With the survey particularly focussing on bariatric surgery, TLS surgeons felt their shoulders and back were mostly affected, whereas RALS surgeons felt their necks were mostly affected, and due to that, a result of reduced caseload and suboptimal performance was observed [24].

Alhusuny et al. [2021] designed a questionnaire with 52 specific items, revealing that TLS modality correlated with vision and neck or shoulder issues, with worsening factors such as forward head movement and uneven weight bearing, whilst it was not found in the RALS modality [25].

## Discussion

Surgeons utilise different muscle groups in TLS and RALS modalities, which both have the potential to cause work-related musculoskeletal disorders. As sEMG is the objective biochemical marker mainly assessing muscle group activity and is used in multiple fields, the reviewed results showed that the deltoid, triceps, biceps and wrist muscles were found to be the main ones used in the TLS, compared to the RALS modality [13, 15, 17, 19, 21]. In the meantime, there were mixed results regarding the muscles activated in the RALS. Lower back, trapezius and finger muscles were activated more and higher in intensity in half of the literature compared [13, 15, 17]. Whilst the RALS modality originally was hypothesised to reduce surgeons’ workload, this comparison showed that the RALS modality is not completely out of muscular straining risks for surgeons. Whilst it relieves some pressures in the arm and shoulder for surgeons, it poses a new risk to the lower back, trapezius and finger muscles, from a biochemical marker standpoint. The differences in findings between studies could also be due to insufficient posture training in surgeons, which might indicate a potential benefit in preoperative warm-ups. Borg CR10 scale is a subjective scale assessing seven body parts, which the surgeons fill out every 30 or 60 min during the surgery. It revealed the same relationship that surgeons felt more fatigue in their lower back in the RALS modality in four studies [12, 13, 22, 23]. Several factors may explain these findings. Marcon et al. [2019] hypothesised that the high Borg score in the lower back in the RALS modality was associated with the fact that surgeons needed to lean forward for the view piece from a sitting position [23]. This effect was diminished as the surgery approached the end when the surgeons were not restricted to the console. Dalsgaard et al. [2020] also noticed that RALS surgeons sat further away from the console as the surgeries went on due to increased concentration and a wheeled chair, leading to a less ergonomic position for surgeons’ lower back and neck [13]. Li-Jen et al. [2020] used a different approach, the Purdue Pegboard test and handgrip test for dexterity and muscular fatigue with a result indicating RALS advantages on general hand muscles [16].

RALS’s advantages on the physical symptoms were also verified on the NASA-TLX survey; however, the surgical modality could be challenging to less experienced surgeons. RALS was shown to reduce the physical demand of the surgery in multiple NASA-TLX studies; however, it required more mental effort and temporal demand, as well as increasing the feeling of frustration in surgeons. It was suggested that most surgeons now are well-trained with TLS, but RALS was still a relatively new modality for surgeons to get familiar with, which could explain why the mental demand and increased effort also disappeared with more years of experience [22]. Surgeons expressed that they were performing better in TLS on the NASA survey from a satisfaction perspective; however, it did not translate to a better patient outcome since there was a study showcasing that patient outcomes are the same in either TLS or RALS [26]. Hence, from a surgeon’s wellbeing standpoint, surgeons should choose the surgical modality best suited for them, since burnout and fatigue correlated with major surgical errors [26].

Muscular activation and fatigue scale are important; however, there are other important factors leading to surgeons’ fatigue. Whilst sEMG provides an objective biochemical measurement of muscle activation and the Borg CR10 scale provides a subjective fatigue level in real-time, surgeons’ posture whilst performing surgeries was only assessed in four studies. REBA and RULA are both validated tools and are used in other industries to assess workers’ posture and individual ergonomics objectively [27]. By continuously rating surgeons’ postures, improvements and areas at risk are identified. Anand et al. [2022] found a moderate deviation of trunk and neck flexion, lower arm flexion and wrist flexion and extension against the recommended degree of angle, which are largely explained by the nature of the robotic console [14]. Dalager et al. [2020] showed that the posture of surgeons also depended on their dominant side; however, RALS generally had a better ergonomic setting than TLS [15]. Dalsgaard et al. [2020] also provided insight into the prolonged length of surgeons abducting their arms, twisting their trunks and asymmetrical weight bearing in TLS, whilst surgeons forgot to utilise existing ergonomic support in RALS in long surgery [13]. Anand et al. [2022] mentioned that with REBA, the surgeons in TLS automatically scored for worse ergonomic postural due to standing up, as contrasted to RALS [14]. For a more accurate assessment, RULA should be utilised in RALS, whilst REBA should be utilised in TLS. Kramer et al. [2022] using gravimetric position sensors also produced similar results [17]. One advantage of using REBA and RULA is that it can be done by any trained observer and provide real-time feedback whilst not disrupting the theatre setting such as sEMG and gravimetric position sensors, with good inter- and intra-reliability [27]. Both sEMG and gravimetric position sensors required the preparation of surgeons’ skin, and the devices required skin-to-skin contact. Whilst it might disrupt the sterile field and limit surgeons’ movements, it also might fall off from surgeons’ skin, leading to inaccurate results.

There are multiple ways to reduce preventable ergonomic risks and unnecessary muscular straining on surgeons when performing MIS. Alhusuny et al. [2021] suggested that as easy as adjusting the height of the operation table and position of the foot pedal and the monitor during TLS could reduce unnecessary trunk twisting and hunching movements [25]. Less than a quarter of RALS surgeons would change into their robotic console ergonomic setting before the surgery began. Dalsgaard et al. [2020] also suggested that using a chair with no wheels could help surgeons reduce trunk flexion in RALS and taking microbreaks can reduce general accumulated fatigue [13]. Lack of awareness still exists amongst surgeons, and better ergonomic training and education are vital to reduce muscular straining in any form of surgery. This indicates the need for future studies focussing on preventative strategies for surgeons, including having warm-up and cool-down before and after the surgery, attention to standing position during a long surgery, and consideration of microbreaks, to ameliorate ergonomic strains.

There are a few limitations in this review. First, simulated tasks were excluded on the basis of including only real-life theatre settings; this unfortunately potentially reduces the data pool analysed within this systematic review. Most of the studies had a low population and some with only one surgeon, impacting the applicability of the findings to general surgeons. Only half of the studies reported their robotic model being Da Vinci, which is a closed platform console, whilst the other half of the studies did not report which robotic model they used. An open platform console would have affected surgeons’ muscular usage profile and ergonomics, prompting a need for multiplatform studies in the future. Although the main validated methods were identified in most of the studies, the muscle groups assessed in sEMG were not standardised and the duration of sEMG measurement was different in each study; thus, the heterogeneity of the methods made it difficult to interpret and compare results. More than half of the studies did not report surgeons’ age and fitness level, which could lead to inaccurate results reported, in both objective and subjective measurements. Even though some studies used the first case of the day for assessment, the baseline of the surgeons’ health should be established. In addition, this study did not look at outcomes across various specialties of surgery.

## Conclusion

In this systematic review, we aimed to characterise the differences in muscle usage, straining patterns and ergonomics in surgeons performing traditional laparoscopic surgery and robotic-assisted laparoscopic surgery. Various methodologies were used in the reviewed articles with different important properties. Our review demonstrates that RALS is a better modality than TLS in terms of muscle straining and ergonomics in surgeons. However, RALS is not completely risk-free, and it requires active surgeon’s input to achieve the best ergonomics and minimise possible damage. Future research on determining an appropriate and constant methodology and exploring the effectiveness of continuous intraoperative ergonomic monitoring is warranted.

## Data Availability

No datasets were generated or analyzed during the current study.

## Abbreviations

MIS: Minimally invasive surgery
TLS: Traditional laparoscopic surgery
RALS: Robotic-assisted laparoscopic surgery
sEMG: Surface electromyography
REBA: Rapid Entire Body Assessment
RULA: Rapid Upper Limb Assessment
NASA-TLX: National Aeronautics and Space Administration Task Load Index

## References

[1] Association IE. What Is Ergonomics [HFE]? Internet https://iea.cc/about/what-is-ergonomics/.

[2] Schlussel AT, Maykel JA (2019) Ergonomics and musculoskeletal health of the surgeon. Clin Colon Rectal Surg 32(6):424–434 31686994 10.1055/s-0039-1693026 PMC6824896

[3] Costa ADS Jr (2017) Assessment of operative times of multiple surgical specialties in a public university hospital. Einstein [Sao Paulo] 15(2):200–205 28767919 10.1590/S1679-45082017GS3902 PMC5609617

[4] Grant KMK, Vo T, Tiong LU (2020) The painful truth: work-related musculoskeletal disorders in Australian surgeons. Occup Med [Lond] 70(1):60–63 10.1093/occmed/kqz155 31829426

[5] Medical Deans Fact Sheet profession entry medical qualifications in australia: contributions and costs for domestic STUDENTS Internet: Medical Deans Australia and New Zealand Inc; 2014 https://medicaldeans.org.au/md/2018/07/2014-July_FACT-SHEETContributions-and-Costs-of-a-Medical-Qualification.pdf.

[6] O'Callaghan J, Mohan HM, Sharrock A, Gokani V, Fitzgerald JE, Williams AP, et al. Cross-sectional study of the financial cost of training to the surgical trainee in the UK and Ireland. BMJ Open. 2017;7[11]:e018086. 10.1136/bmjopen-2017-018086 PMC5695344 29146646

[7] Agha R, Muir G (2003) Does laparoscopic surgery spell the end of the open surgeon? Jrsm 96(11):544–546 14594961 10.1258/jrsm.96.11.544 PMC539626

[8] Palep JH (2009) Robotic assisted minimally invasive surgery. J Minim Access Surg 5(1):1–7 19547687 10.4103/0972-9941.51313 PMC2699074

[9] Alleblas CCJ, de Man AM, van den Haak L, Vierhout ME, Jansen FW, Nieboer TE (2017) Prevalence of musculoskeletal disorders among surgeons performing minimally invasive surgery: a systematic review. Ann Surg 266(6):905–920 28306646 10.1097/SLA.0000000000002223

[10] Alnefaie MN, Alamri AA, Hariri AF, Alsaad MS, Alsulami AM, Abbas AM et al (2019) Musculoskeletal symptoms among surgeons at a tertiary care center: a survey based study. Med Arch 73(1):49–54 31097861 10.5455/medarh.2019.73.49-54 PMC6445622

[11] Stucky CH, Cromwell KD, Voss RK, Chiang YJ, Woodman K, Lee JE et al (2018) Surgeon symptoms, strain, and selections: systematic review and meta-analysis of surgical ergonomics. Ann Med Surg [Lond] 27:1–8 10.1016/j.amsu.2017.12.013 PMC5832650 29511535

[12] Hotton J, Bogart E, Le Deley MC, Lambaudie E, Narducci F, Marchal F (2023) Ergonomic assessment of the surgeon’s physical workload during robot-assisted versus standard laparoscopy in a french multicenter randomized trial [ROBOGYN-1004 Trial]. Ann Surg Oncol 30(2):916–923 36175710 10.1245/s10434-022-12548-3

[13] Dalsgaard T, Jensen MD, Hartwell D, Mosgaard BJ, Jorgensen A, Jensen BR (2020) Robotic surgery is less physically demanding than laparoscopic surgery: paired cross sectional study. Ann Surg 271(1):106–113 29923873 10.1097/SLA.0000000000002845

[14] Anand S, Sandlas G, Pednekar A, Jadhav B, Terdal M. A Comparative Study of the Ergonomic Risk to the Surgeon During Vesicoscopic and Robotic Cross-Trigonal Ureteric Reimplantation. J Laparoendosc Adv Surg Tech A. 2021. 10.1089/lap.2021.0471 34449268

[15] Dalager T, Jensen PT, Eriksen JR, Jakobsen HL, Mogensen O, Sogaard K (2020) Surgeons’ posture and muscle strain during laparoscopic and robotic surgery. Br J Surg 107(6):756–766 31922258 10.1002/bjs.11394

[16] Kuo LJ, Ngu JC, Lin YK, Chen CC, Tang YH. A pilot study comparing ergonomics in laparoscopy and robotics: beyond anecdotes, and subjective claims. J Surg Case Rep. 2020;2020[2]:rjaa005. 10.1093/jscr/rjaa005 PMC7033486 32104561

[17] Kramer B, Neis F, Reisenauer C, Walter C, Brucker S, Wallwiener D, et al. Save our surgeons [SOS] - an explorative comparison of surgeons' muscular and cardiovascular demands, posture, perceived workload and discomfort during robotic vs. laparoscopic surgery. Arch Gynecol Obstet. 2023;307[3]:849–62. 10.1007/s00404-022-06841-5 PMC9676911 36401096

[18] Butler KA, Kapetanakis VE, Smith BE, Sanjak M, Verheijde JL, Chang YH et al (2013) Surgeon fatigue and postural stability: is robotic better than laparoscopic surgery? J Laparoendosc Adv Surg Tech A 23(4):343–346 23410117 10.1089/lap.2012.0531

[19] Zihni AM, Ohu I, Cavallo JA, Cho S, Awad MM (2014) Ergonomic analysis of robot-assisted and traditional laparoscopic procedures. Surg Endosc 28(12):3379–3384 24928233 10.1007/s00464-014-3604-9

[20] Szeto GP, Poon JT, Law WL (2013) A comparison of surgeon’s postural muscle activity during robotic-assisted and laparoscopic rectal surgery. J Robot Surg 7(3):305–308 27000928 10.1007/s11701-012-0374-z

[21] Armijo PR, Huang CK, High R, Leon M, Siu KC, Oleynikov D (2019) Ergonomics of minimally invasive surgery: an analysis of muscle effort and fatigue in the operating room between laparoscopic and robotic surgery. Surg Endosc 33(7):2323–2331 30341653 10.1007/s00464-018-6515-3

[22] Mendes V, Bruyere F, Escoffre JM, Binet A, Lardy H, Marret H et al (2020) Experience implication in subjective surgical ergonomics comparison between laparoscopic and robot-assisted surgeries. J Robot Surg 14(1):115–121 30863913 10.1007/s11701-019-00933-2

[23] Marcon B, Ngueyon Sime W, Guillemin F, Hubert N, Lagrange F, Huselstein C et al (2019) An ergonomic assessment of four different donor nephrectomy approaches for the surgeons and their assistants. Res Rep Urol 11:261–268 31579673 10.2147/RRU.S220219 PMC6773969

[24] AlSabah S, Al Haddad E, Khwaja H (2019) The prevalence of musculoskeletal injuries in bariatric surgeons. Surg Endosc 33(6):1818–1827 30251136 10.1007/s00464-018-6460-1

[25] Alhusuny A, Cook M, Khalil A, Johnston V (2021) Visual symptoms, neck/shoulder problems and associated factors among surgeons performing Minimally Invasive Surgeries [MIS]: a comprehensive survey. Int Arch Occup Environ Health 94(5):959–979 33515063 10.1007/s00420-020-01642-2

[26] Grochola LF, Soll C, Zehnder A, Wyss R, Herzog P, Breitenstein S (2019) Robot-assisted versus laparoscopic single-incision cholecystectomy: results of a randomized controlled trial. Surg Endosc 33(5):1482–1490 30218263 10.1007/s00464-018-6430-7

[27] Kee D. Systematic Comparison of OWAS, RULA, and REBA Based on a Literature Review. Int J Environ Res Public Health. 2022;19[1]. 10.3390/ijerph19010595 PMC8744662 35010850

[28] Tarr ME, Brancato SJ, Cunkelman JA, Polcari A, Nutter B, Kenton K (2015) Comparison of postural ergonomics between laparoscopic and robotic sacrocolpopexy: a pilot study. J Minim Invasive Gynecol 22(2):234–238 25315401 10.1016/j.jmig.2014.10.004

